# The Hahnemannian’s Analysis Sheet

**Published:** 1891-05

**Authors:** M. A. A. Wolff

**Affiliations:** Gainesville, Texas


### THE HAHNEMANNIAN'S ANALYSIS SHEET.

GAINESVILLE, TEXAS, April 9th, 1891.

EDITORS OF THE HOMŒOPATHIC PHYSICIAN:

In the April issue of THE HOMŒOPATHIC PHYSICIAN, I see a letter from Dr. Heath, of London, Eng., in which he seemingly accuses me of plagiarism. Another physician sent me a copy of his "Repertory checking list." Before I adopted my new plan I used something like this, viz., sheets printed from my "label-plate." Dr. Lee's plan I do not know where to look for, but from your remark that my plan is a "modification" of it I glean that so far I am not considered a plagiarist. Neither can I, by studying Dr. Heath's case in the *Advance* of December, 1891, find it to be so. But "original," as my plan will be to me until I am shown its equal antedating mine, or not—I do not care. It is not the honor of originality for which I here contend, it is the accusation of plagiarism I want to refute. For a time I used my sheet only for myself, its nearest, "Guernsey's Bœnninghausen slips," at which I had jumped with enthusiasm, soon proving themselves too cumbersome. Seeing how well my plan worked, I had plates made and had it printed for the benefit of the fraternity. The points which make the analysis sheet specially useful (and it is in this I claim its originality) are: You have not to arrange your symptoms but look them up and mark them off from the repertory just as they happen in the patient's report. Then there is an upper and lower rubric for each remedy, the one for the values 1 and 2, the other for 3 and 4, indicating the difference by whole or half strokes, and if very particular these strokes may be light or heavy, and thus at a glance you will see as well the number as the importance. Lastly, the same sheet is good through the whole treatment, having room for patient's name, etc., date of prescription, directions, and further prescriptions until you are through. If anything like it, or rather, equal to it, has been published before, I shall gladly let the author enjoy the laurels, for all I care for is that it shall be useful to all concerned. Hahnemann did not invent Homœopathy, but he nailed it down as a science. If I did not first invent the plan bespoken, I do not care, it is the way to use it—I believe—that I have made public.

Yours truly,

M. A. A. Wolff.

